# Access to quality healthcare for trans people

**DOI:** 10.1038/s43856-023-00316-7

**Published:** 2023-06-28

**Keywords:** Public health, Health policy

## Abstract

Dr. Kamilla Kamaruddin is a general practitioner in Transgender Health Care and Clinical Lead at the East of England Gender Service, Cambridge, UK. She is also a board member for sexual health and wellbeing organisation Spectra-London, trustee at LGBTQ+ cancer charity Live Through This, and health advisor for trans community organisation TransActual UK. In this Q&A, we ask Dr. Kamaruddin a series of questions on the difficulties transgender people face in accessing quality healthcare, with a particular focus on the UK.

**Figure:**
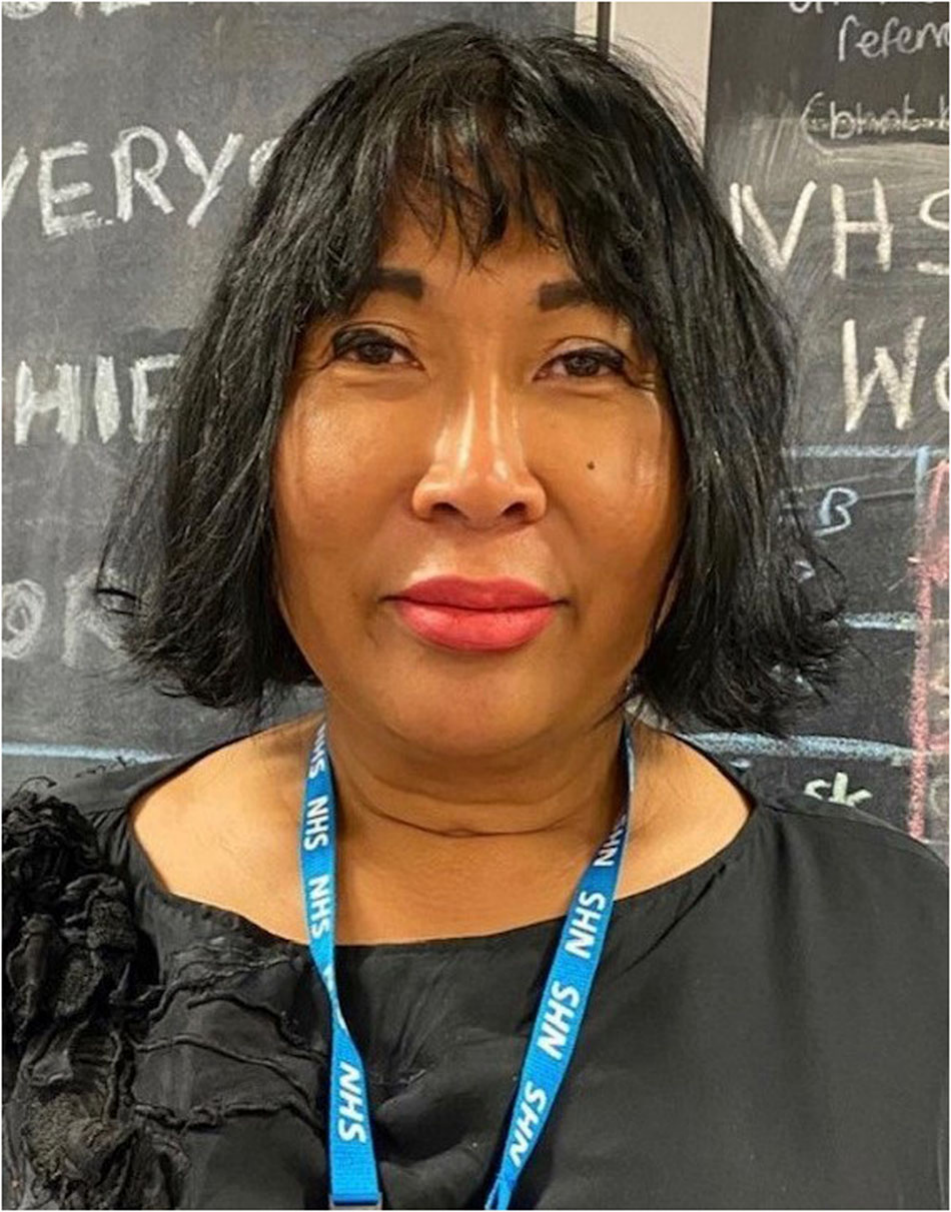
Kamilla Kamaruddin

What kind of challenges do transgender people face in accessing the healthcare that they need?

Transgender (trans) people face multiple barriers and prejudices when accessing healthcare, both in terms of access to gender services and healthcare more generally. In a report on the health of LGBT people in Britain by the LGBTQ+ charity Stonewall published in 2018^1^, 62% of trans people accessing general healthcare services said, when surveyed, that staff lacked understanding of specific trans health needs, while 16% said they had been refused healthcare because they are LGBT. The survey also identified a substantial burden of poor mental health amongst trans people. Forty-six percent of trans people had thought about taking their own life in the previous year. In my experience, healthcare professionals have a lack of awareness of the barriers that exist in healthcare for trans and gender-diverse people and how this impacts them.

There are many kinds of barriers preventing trans people from accessing healthcare, including structural, educational, technical, and societal ones^2^. In the UK, there is a shortage of gender services, and many general practitioners (GPs) are reluctant to be involved in trans healthcare. The shortage of gender services and clinicians trained in trans health means that there are long waiting times to be seen by gender services, with average waiting times being 2 to 3 years. At present, it is estimated that there are 20,000 people waiting to be seen in the UK. There is also a lack of gender-affirming services, which provide a range of social, psychological, and medical interventions to support an individual’s gender identity, for trans and gender-diverse children and adolescents, and a lack of trans-specific mental health services more broadly. When accessing health services, trans people can face prejudice in healthcare and microaggressions – subtle verbal or non-verbal insults or indignities that may or may not be intentional - that can impact their wellbeing. A systemic lack of clinical training, education, and awareness on trans health are some of the contributing factors^3^, but these are issues that could easily be overcome. Another challenge is one of a technical nature, which is a lack of demographic data within patient records to identify trans people that can make it difficult for health professionals to provide good medical care. Also, the feeling that trans people are routinely portrayed in a negative light in the UK media, compounded with the fact they are a minority, makes it very difficult for trans people, and in particular for children, their parents, and families, to seek the support and healthcare that they need. So, even if care is available, there are additional barriers preventing trans people from accessing it.

What do you think are the reasons underlying these issues?

One key reason underlying these issues is a historical lack of funding and resourcing for healthcare for trans people. Trans healthcare is still inadequately funded and substantial investment is needed in clinical training and infrastructure to reduce inequalities and, in particular, to reduce the amount of time trans people have to wait to access gender services. Fuelling these inequalities is the pushback trans people feel against their right to exist in the UK media, which is perceived by many as relentless, discriminatory, and as a result diminishes their sense of belonging in UK society. This leads to stigma which makes it harder for trans people to feel comfortable in seeking support. Trans people have existed since the dawn of time and have contributed to society, yet we are made to feel unworthy. Many trans people also feel that society’s institutions are inherently unsupportive of trans people, or use anti-trans rhetoric, in a way that can also harm their mental health. There are support organisations and advocacy groups that can support trans people in the community, but they do not receive adequate and sustainable funding.

How might the challenges faced by a trans person in the UK differ from those experienced by a trans person elsewhere?

The long waiting lists are similar in many Western European countries and trans people face huge challenges to access medical and surgical transition. In many European countries, trans people also require a diagnosis from a psychiatrist, pathologizing their gender identity. Some European countries require sterilisation before trans people can change their preferred name and gender on official documents and, in my opinion, this is a cruel practice rooted in the idea that being transgender is a psychiatric illness. Trans people should have the right to preserve their fertility. In the UK, trans people can self-declare and change their name and title on their formal documents with a deed poll certificate. A gender recognition certificate can be obtained which allows trans people to apply for a birth certificate that aligns with their gender identity.

The lack of resources, funding, education, and training on trans healthcare we have in the UK is similar in many other countries. Conversion therapy, which attempts to change a person’s sexual orientation or gender identity, is not banned for trans people in the UK despite evidence that this practice is harmful to their mental health. France, Germany, Holland, and Belgium have banned conversion therapy or are in the process of implementing bans. In many other countries outside Europe, trans people are even denied their safe existence, let alone given access to healthcare and education. Trans people are still subjected to discrimination, prejudice, violence and even death in some parts of the world just for being who they are. Trans visibility and the opportunity to live their lives authentically is a privilege that many trans people cannot yet have around the world.

What can individual clinicians do to better serve the trans community?

The humility and willingness from a healthcare professional to learn about healthcare for trans people can be an enormous source of comfort and support. In my opinion, individual clinicians have a duty to improve the wellbeing of trans people and should always focus on what would be in the best interests of the patients, who in this case happen to be trans. There are advice and guidance services available from all gender services in the UK if a clinician wishes to seek advice about treating trans people or has any queries related to gender. Resources are also widely available from Gender Identity Clinics’ websites and the Royal College of General Practitioners LGBT e-learning hub, which is also available for non-members. Increased availability of these resources will help clinicians build knowledge and competency in treating trans people. If any NHS organisation wishes to access training on trans issues and care, I am always available to provide advice via email in my capacity as a GP in Transgender Health Care and Clinical Lead at the East of England Gender Service.

What broader changes are needed in healthcare systems and health policy to provide quality healthcare for trans people?

Teaching on LGBTQ+ health should be made a more structured part of the medical curriculum for medical students and trainee doctors. At present, LGBTQ+ teaching is sporadic, voluntary, and inadequate. NHS trusts should invest in better IT systems and electronic health records for gender services to better collect data, measure outcomes, demographics, and performance of services. This can help gender specialists in providing better healthcare for trans people and improve efficiency. Within general practice, medical records are not set up in a way that makes it easy for GPs to identify trans people and provide high quality patient-centred care. Other aspects of trans healthcare, such as cancer screening, also need to be improved. At present, trans people with a cervix do not get an automatic invite for cervical cancer screening. As more GPs and nurses become involved with providing care to trans people, it would be helpful for the NHS to open more GP- and nurse-led gender services. Nurses play a key role in the provision of holistic care to trans people, easing clinical workloads, and improving patient satisfaction. At present, there are only four pilot gender services in the UK, in Liverpool, Manchester, London, and Cambridge. Gender services are collaborating with other health professionals in secondary care to improve healthcare for trans people. For example, I am collaborating with Royal College of Anaesthetists to create national guidelines for perioperative care for trans people^4^.

What role do advocacy organisations play in improving healthcare for trans people?

Many trans people have a good experience when engaging with charitable organisations and trans support groups^5^. These organisations can help to provide safe meeting spaces, host trans-led support groups, improve fitness and wellbeing, improve mental health by providing trans-specific counselling sessions, support trans people affected by cancer, support trans people in their social transition, provide information about trans-specific sexual health clinics, give employment advice, and improve social cohesion by building self-confidence. Advocacy organisations can have an enormous impact on physical and mental health in the long term, helping to reduce health inequalities faced by trans people and helping them reach their full potential by being empowered to make positive, fully informed life choices.
